# Recombinant Platelet-Derived Growth Factor BB vs Autologous Nanofat to Enhance Recovery After CO_2_ Laser and Microneedling: A Split-Face, Randomized Controlled Trial

**DOI:** 10.1093/asjof/ojag033

**Published:** 2026-03-06

**Authors:** R Brannon Claytor, Patricia M Fuentes, Grace Tolan, Kay Durairaj, Robert Quigley, Samuel E Lynch

## Abstract

Nanofat has been used as a “rescue” modality to support healing after CO_2_ laser resurfacing with microneedling. Because demand grows for less invasive facial rejuvenation options, recombinant platelet-derived growth factor (rhPDGF-BB) has emerged as a popular adjunct to enhance postresurfacing recovery. The aim of this study was to compare autologous Nanofat to rhPDGF-BB as rescue treatments for pain and skin recovery following combined thermal and mechanical injury for improvement of facial rhytids. Five patients underwent full-face CO_2_ laser treatment followed by microneedling. Each patient received split-face randomization: one side was treated with Nanofat and the contralateral side with rhPDGF-BB (300 µg/mL). Patients were blinded to allocation. Standardized 3 mm biopsies were obtained at baseline and at postprocedure Day 4, 1 month, 3 months, and 6 months and evaluated by a blinded board-certified histopathologist. Outcomes included practitioner- and patient-reported Perioral Rhytid Severity Rating Scale (PR-SRS), Global Aesthetic Improvement Scale (GAIS), and patient satisfaction. Both treatments improved patient/practitioner PR-SRS and GAIS beginning on Day 4 and continuing through 6 months. There were no significant differences between treatment groups (*P* > .05). Patients reported high satisfaction with both treatment groups. Overall, histopathology assessments demonstrated that both Nanofat and rhPDGF-BB supported normal wound healing and produced comparable tissue remodeling over time. Differences between treatments were subtle and confined to the early inflammatory interval, with Nanofat showing a consistently milder inflammatory response. This is the first human histologic split-face study of rhPDGF-BB in facial rejuvenation. This preliminary study found that either Nanofat or rhPDGF-BB treatment following full-face CO_2_ laser treatment and microneedling resulted in improved clinical and histologic outcomes. Nanofat showed milder early inflammatory changes, but both treatments produced normal wound healing and robust extracellular matrix formation and comparable tissue remodeling by 1, 3, and 6 months.

**Level of Evidence:** 2 (Therapeutic) 
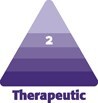

Regenerative aesthetic medicine is a rapidly expanding field, offering a variety of products with the goal of targeting the visible signs of aging and photodamaged skin. There is a growing demand from practitioners and patients for innovative and minimally invasive approaches for skin rejuvenation that prioritize safety, efficacy, and durability of aesthetic results. This rapidly evolving field aims to utilize biochemical modulators, strengthen extracellular properties to restore skin elasticity, modulate inflammatory pathways, and optimize cellular signals to drive tissue and cellular regeneration.^1-3^

Aging, photodamaged skin is characterized by dermal atrophy, loss of elasticity, rhytid formation, and the appearance of fine “crepe” texture. Underlying these visible characteristics are extensive biochemical and cellular changes to the epidermis, dermis, and extracellular matrix of the skin. Specifically, the formation of rhytids involves a complex cascade resulting in loss of tensile strength, dermal fibrosis, impaired cellular signaling, and reduced cellular repair capacity.^3-5^

To target the inevitable changes of aging and photodamaged skin, various energy-based devices and mechanical interventions, such as CO_2_ lasers and microneedling, are readily utilized to induce controlled dermal trauma, thus stimulating cellular regeneration and collagen synthesis. Ablative CO_2_ lasers stimulate skin remodeling by triggering neo-collagenesis as well as vaporizing aged epidermis and inducing skin tightening.^6^ Similarly, mechanical microneedling induces micro-injury within the papillary and reticular dermis, which stimulates collagen and elastin synthesis through minimally invasive percutaneous collagen induction. By creating micro-punctures into the skin, microneedling allows for the immediate delivery of adjunct products such as autologous Nanofat and growth factors to accelerate recovery.^7-9^ A previous study reported that aggressive fractional CO_2_ laser with immediate subsequent mechanical microneedling—which historically had been considered too injurious to perform simultaneously—can effectively be rescued with autologous Nanofat thus resulting in an accelerated recovery and significant reduction in postprocedure pain.^10^

In current practice, incorporating a rescue element typically requires harvesting lipoaspirate for autologous Nanofat or venipuncture for preparation of platelet-rich plasma (PRP). These approaches are labor intensive and operator dependent and may result in wound complications and/or donor-site morbidity. Autologous Nanofat delivers a mixture of adipose-derived stem cells (ASCs), extracellular matrix proteins, and lipid droplets that contain cytokines and mRNA.^11-14^ PRP is a source of growth factors, including platelet-derived growth factor (PDGF), vascular endothelial growth factor (VEGF), epidermal growth factor (EGF), and fibroblast growth factor, as well as cytokines, but the resultant preparation can vary widely between patients and the effect can be concentration dependent.^3,15-17^

Recombinant PDGF-BB is FDA approved to improve healing of full thickness skin wounds in diabetics, and improve tissue regeneration following oral maxillofacial surgery and orthopedic surgery.^18,19^ It has recently been introduced to medical aesthetics to provide a more potent and more consistent source of growth factors compared with other biological preparations (ie, PRP, recombinant EGF, platelet-rich fibrin, etc). The potential to improve PDGF-mediated wound healing during facial skin rejuvenation procedures may now be utilized by commercially available on-demand recombinant PDGF. PDGF comprises 5 isoforms (AA, BB, AB, CC, and DD) and is a potent chemoattractant for dermal fibroblasts, stem cells, smooth muscle cells, and endothelial cells.^20^ PDGF-BB, the most potent form of PDGF, has been shown to play a key role in wound healing by recruiting cells responsible for healing, stimulating extracellular matrix synthesis, and promoting angiogenesis through VEGF signaling.^19^

Although Nanofat is widely used for wound healing during facial skin rejuvenation procedures, rhPDGF-BB is a new and compelling alternative for skin rejuvenation.^21-24^ Unlike currently available biologics, rhPDGF-BB is laboratory engineered, thus offering consistent growth factor concentrations, and does not require venipuncture or liposuction. Its established chemotaxis, mitogenesis, and angiogenic properties may have the potential for improved wound healing in skin rejuvenation.

Therefore, the objective of this split-face, randomized controlled clinical trial is to evaluate the safety and efficacy of rescue rhPDGF-BB compared to autologous Nanofat following thermal injury CO_2_ fractional laser and mechanical trauma from microneedling for skin facial rejuvenation and including an assessment of pre- and posttreatment histopathological changes using facial tissue biopsies.

## METHODS

**Figure 1.**
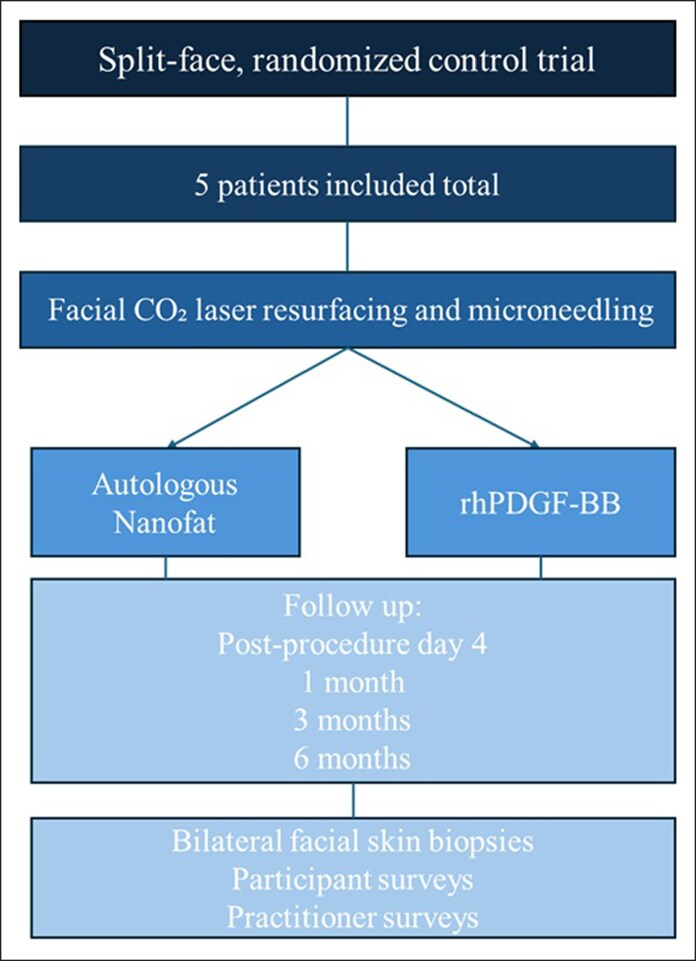
Protocol outline and procedures conducted on each visit. Five patients were included in this split-face, randomized controlled trial where autologous Nanofat was randomized to one half of the face and recombinant platelet-derived growth factor (rhPDGF-BB) alone (without hyaluronic acid) was randomized to the contralateral half of the face after CO_2_ laser resurfacing and microneedling.

Five patients were recruited and enrolled in the study between October 2024 and September 2025. IRB approval was granted by Advarra Institutional Review Board Pro00081971 on September 13, 2024. The principles of the Declaration of Helsinki were adhered to in this study. Written informed consent was provided by all patients who agreed to participate in the study for the use of their data for analysis (Figure 1).

### Study Products

In this study, autologous Nanofat was compared to recombinant human PDGF-BB (rhPDGF-BB; Ariessence Pure PDGF, LRM Aesthetics, Franklin, TN). Autologous Nanofat was selected as the comparator rather than a no-treatment control based on existing evidence demonstrating its safety and efficacy in skin quality improvement.^10,22,25,26^

### Patient Recruitment and Randomization

Eligible patients included adults 30 to 90 years of age with Fitzpatrick skin types I to VI who were willing to comply with all study visits, refrain from additional procedures to the treatment area during the study period, and provide written consent. Patients recruited were concerned with facial rhytids. Exclusion criteria included current participation in another investigational drug or device study or recent investigational treatment to the study area within 3 months, previous gold thread skin rejuvenation, active wounds or infection in the treatment area, allergy to adhesives or topical anesthetics, nerve insensitivity to heat in the treatment area, severe skin laxity or redundant tissue that would limit treatment efficacy, and isotretinoin use within the previous 6 to 12 months.

Randomization was performed at the time of the procedure using a coin-flip method. Heads corresponded to autologous Nanofat (“Nanofat” group) and tails to rhPDGF-BB (“PDGF” group). The coin flip determined treatment allocation to the right hemiface, with the contralateral side receiving the alternate treatment.

### Procedure

All patients were treated by the primary author and surgeon (R.B.C.) under local anesthesia with topical benzocaine 20%, lidocaine 10%, and tetracaine 10% with supplemental infraorbital and mental nerve blocks with 1% lidocaine administered at the primary surgeon's discretion. All patients underwent CO_2_ laser treatment (DEKA Tetra SmartXide CO_2_, Calenzano, Italy) in 2 applications to the designated treatment area. The first application was performed in DEKA pulse setting (25-30 W, dwell time of 500 µs, spacing of 500 µm, for 13% coverage). This application was performed for the entire face, including periorbital and retroauricular regions. The subsequent application was in the high pulse setting (4.0-5.0 W, spacing of 100 µm, and 100% coverage) along the same areas. Immediately following CO_2_ laser treatment, the same area was treated with microneedling (SkinPen, Crown Aesthetics, Dallas, TX) with the depth set to 2.5 mm. Nanofat was harvested from autologous lipoaspirate from the abdomen. A 2 mm cannula with hand liposuction was used. Dependent on patient BMI, a solution of 500 mL normal saline, 1 mg epinephrine (1:1000), and 20 mL of 1% lidocaine was infiltrated into the abdominal skin.

Following combined CO_2_ laser and microneedling treatment, the treatment area was divided into 2 halves. By random allocation, one half of the face was treated with topical application of sterile rhPDGF-BB and the contralateral half was treated with freshly harvested autologous Nanofat. rhPDGF-BB consisted of a sterile aqueous solution containing 99.7% water, 0.27% sodium acetate, and 0.03% SH-Polypeptide-59 (rhPDGF-BB). Only the rhPDGF-BB solution was used (ie, the hyaluronic acid [HA] that is also packaged in the product was not used). A total volume of 2.5 mL was applied topically in droplets through a syringe, and microneedling was performed until all of the rhPDGF-BB solution was absorbed.

Autologous Nanofat was harvested, using sterile technique, from autologous lipoaspirate and processed sequentially through 2.4, 1.4, and 1.2 mm connectors for 20 sequential back-and-forth passes, then it was filtered through a 500-micron filter to produce a total of 20 cc autologous nanofat (Tulip Medical, San Diego, CA).^27^ A total volume of 2.5 mL autologous Nanofat was similarly applied, and microneedling was performed until fully absorbed.

### Postprocedure Care

Following treatment, patients were instructed to apply Soothe + Protect Recovery Balm (Alastin Skincare, Inc., Carlsbad, CA) to the treated areas approximately every hour (up to 16 applications per day) and Regenerating Skin Nectar (Alastin Skincare, Inc.) every 12 h for 7 days. Patients were advised to avoid the use of cosmetics, makeup, or harsh cleansers on the treated areas for at least 24 h postprocedure. Patients were instructed to reasonably avoid direct sun exposure, and applying broad-spectrum sunscreen with SPF ≥30 was recommended for daily use beginning 24 h after treatment. Patients were prescribed oxycodone (5 mg) and diazepam (15 mg) for up to 2 days, to be taken every 4 h as needed for pain control.

### Rating Scales

Patient demographics, skin characteristics, procedural details, and outcomes were recorded. Intraoperative pain was recorded using a 0 to 10 numeric rating scale.^28^ Hemiface pain scores were collected for time zero (immediately after the procedure and application of treatment) and 5 min postprocedure. Participants completed pain diaries immediately postprocedure and at 6 h, 24 h, 1 week, 2 weeks, and 1 month for the lipoaspirate donor site (LDS) and global face. Follow-up visits were scheduled for 4 days, 1 month, 3 months, and 6 months postprocedure. At each visit, the participants completed a patient satisfaction survey and Perioral Rhytid Severity Rating Scale (PR-SRS). The participants were blinded to the treatment allocated to each hemiface. Concurrently, the primary surgeon completed a Global Aesthetic Improvement Scale (GAIS) Assessment and PR-SRS for each half of the face. PR-SRS surveys will be differentiated as “patient-reported PR-SRS” and “practitioner-reported PR-SRS.” All surveys are included in Appendices A and B.

### Tissue Biopsy

All patients underwent serial punch biopsies in the postauricular region at baseline prior to treatment and then at 4 days, 1 month, 3 months, and 6 months postprocedure for histopathologic evaluation. The biopsy site was predetermined and marked bilaterally. All specimens were sent for hematoxylin and eosin (H&E) and trichrome stains. All histological slides were sent for blinded histopathological descriptive analysis by a third-party board-certified dermatopathologist (Lanterne Dx, Boulder, CO).

### Statistical Analysis

All statistical analyses were performed using IBM SPSS version 30.0 (IBM Corp., Armonk, NY). Between-treatment comparisons between the Nanofat- and rhPDGF-BB treated hemifaces at each time point were performed using paired Wilcoxon signed-rank tests. Within-treatment longitudinal comparisons were also performed using paired Wilcoxon signed-rank tests, comparing follow-up time points to baseline values for patient-reported PR-SRS and practitioner-reported PR-SRS outcomes. For outcomes in which baseline values were not collected (practitioner-reported GAIS and patient satisfaction), within-treatment comparisons were performed relative to postprocedure day (POD) 4 values. Statistical significance was set at *P* < .05.

## RESULTS

### Demographic Characteristics

Five patients, all female, volunteered to participate in the study from October 2024 to September 2025. The mean patient age was 62.8 years (range, 54-71 years). Patient 2 had a history of facelift 2 years prior to the study. Four patients were Fitzpatrick skin type 1, and 1 patient was Fitzpatrick skin type 2.

### Pain Scores

**Figure 2.**
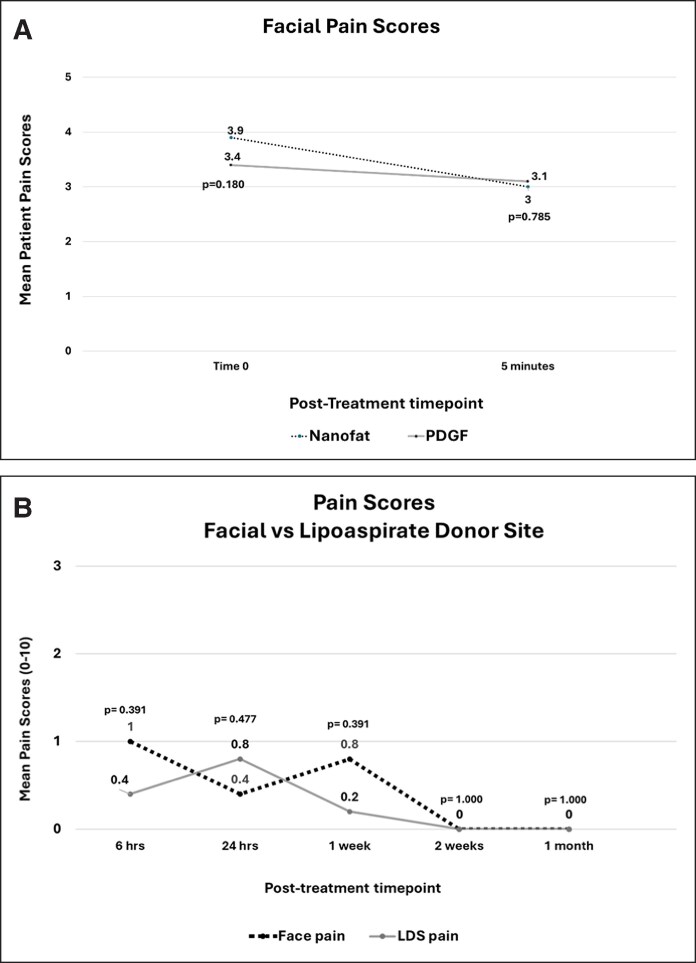
The mean pain scores with possible scores from 0 to 10. (A) The mean hemiface pain scores of the autologous nanofat- and recombinant platelet-derived growth factor (rhPDGF-BB)-treated sides. There were no differences in the mean pain scores for the autologous nanofat- and rhPDGF-BB-treated sides immediately after the procedure (Time 0) and 5 min after the procedure. (B) The mean global face and lipoaspirate donor-site (LDS) pain scores. There were no differences in pain scores between the face and LDS at 6 h, 24 h, 1 week, 2 weeks, and 1 month after the procedure.

One patient reported using diazepam as indicated for pain control on the day of the procedure. No other patients reported using the prescribed medications (oxycodone or diazepam). All pain scores were reported using a rating scale of 0 to 10.^28^ At time 0, the mean pain scores were 3.9 ± 2.51 for the Nanofat-treated hemiface and 3.4 ± 2.07 for the PDGF-treated hemiface (*P* = .180; Table 1, Figure 2). At 5 min posttreatment, the mean pain scores were 3.0 ± 1.87 for the Nanofat-treated hemiface and 3.1 ± 1.88 for the PDGF-treated hemiface (*P* = .785).

**Table 1.** Comparison of Outcomes Between Autologous Nanofat and rhPDGF-BB at All Study Time Points Including PR-SRS (Patient and Practitioner), Practitioner GAIS, Patient Satisfaction Scores, and Hemiface Pain Scores

Outcome	Time point	Nanofat	PDGF	*P*-value
Patient PR-SRS	Baseline	2.40 ± 0.89	2.40 ± 0.89	1.000
	POD 4	0.60 ± 0.55	0.60 ± 0.55	1.000
	1 month	1.00 ± 0.00	1.00 ± 0.00	1.000
	3 months	1.40 ± 0.89	1.40 ± 0.89	1.000
	6 months	0.80 ± 0.45	0.80 ± 0.45	1.000
Practitioner PR-SRS	Baseline	2.50 ± 0.58	2.50 ± 0.58	1.000
	POD 4	0.00 ± 0.00	0.00 ± 0.00	1.000
	1 month	0.50 ± 0.58	0.75 ± 0.50	.317
	3 months	0.75 ± 0.50	1.00 ± 0.00	.317
	6 months	0.75 ± 0.50	0.75 ± 0.50	1.000
Practitioner GAIS	POD 4	1.25 ± 0.50	1.50 ± 1.00	.317
	1 month	1.00 ± 0.00	1.25 ± 0.50	.317
	3 months	1.00 ± 0.00	1.25 ± 0.50	.157
	6 months	1.00 ± 0.00	1.00 ± 0.00	1.000
Patient satisfaction	POD 4	5.00 ± 0.82	5.25 ± 0.50	.317
	1 month	5.00 ± 1.41	5.00 ± 1.41	1.000
	3 months	5.75 ± 0.50	5.75 ± 0.50	1.000
	6 months	6.00 ± 0.00	6.00 ± 0.00	1.000
Pain	Time 0	3.90 ± 2.51	3.40 ± 2.07	.180
	5 min	3.00 ± 1.87	3.10 ± 1.88	.785

GAIS, Global Aesthetic Improvement Scale; PDGF, platelet-derived growth factor; POD, postoperative day; PR-SRS, Perioral Rhytid Severity Rating Scale; rhPDGF-BB, recombinant platelet-derived growth factor.

Pain at the LDS and global face was evaluated immediately postprocedure and at serial intervals (6 h, 24 h, 1 week, 2 weeks, and 1 month). Immediately postprocedure, the mean pain score for the face was 2.6 ± 1.14, and for the LDS donor site, it was 0.2 ± 0.45 (*P* = .042). At 6 h, the mean pain score for the face was 1.25 ± 1.50 (*n* = 4), and for the LDS donor site, it was 0.5 ± 0.58 (*P* = .276). At 24 h, the mean pain score for the face was 0.4 ± 0.89 (*n* = 5), and for the LDS donor site, it was 0.8 ± 0.84 (*P* = .414). At 1 week, the mean pain score for the face was 0.25 ± 0.50 (*n* = 4), and for the LDS donor site, it was 0.00 ± 0.00 (*P* = .317). At 2 weeks, the mean pain score for both the face and LDS donor site was 0.00 ± 0.00 (*P* = 1.000). At 1 month, the mean pain score for both the face and LDS donor site was 0.00 ± 0.00 (*P* = 1.000).

### Patient-Reported PR-SRS

**Figure 3.**
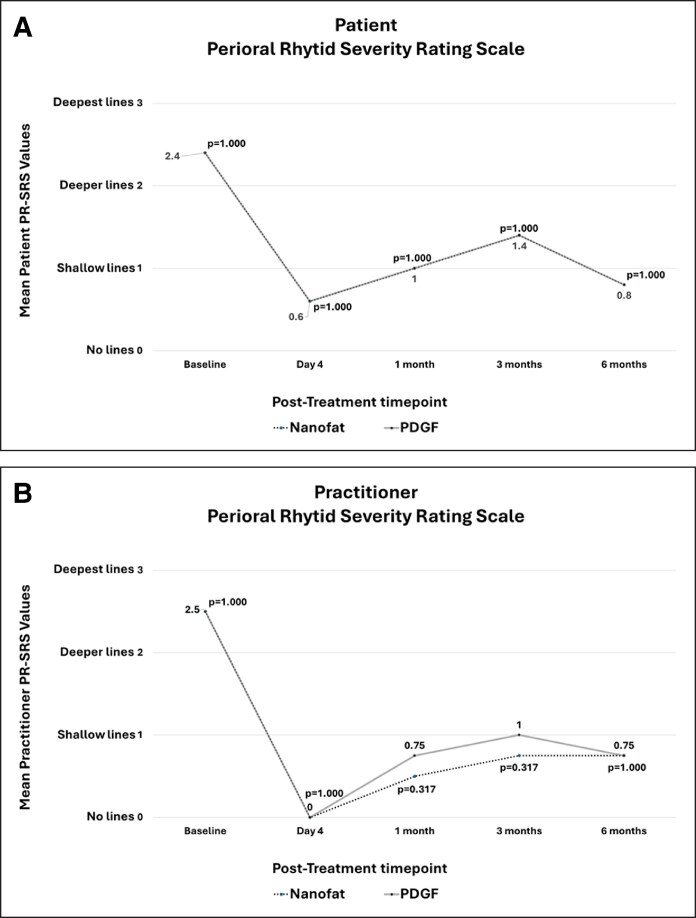
Perioral Rhytids Severity Scale (PR-SRS) Assessment with possible scores of 0 to 3. A score of 0 represents absent perioral lines and a score of 3 represents severe, deepest perioral lines. There was no significant difference in perioral rhytid mean scores between the Nanofat- and PDGF-treated sites (all *P* > .05). (A) Patient-reported PR-SRS mean values. (B) Practitioner-reported PR-SRS mean values.

At baseline, the mean PR-SRS scores were 2.4 ± 0.89 for both the Nanofat and PDGF sides (*P* = 1.000; Figure 3). On POD 4, the mean scores were 0.6 ± 0.55 for both hemifaces (*P* = 1.000). At 1 month, the mean scores were 1.0 ± 0.00 for both hemifaces (*P* = 1.000). At 3 months, the mean scores were 1.4 ± 0.89 for both hemifaces (*P* = 1.000). At 6 months, the mean scores were 0.8 ± 0.45 for both hemifaces (*P* = 1.000).

After a within-nanofat comparison to baseline, at POD 4, the mean PR-SRS was 0.6 ± 0.55 (*P* = .066 vs baseline). At 1 month, the mean PR-SRS was 1.0 ± 0.00 (*P* = .059 vs baseline). At 3 months, the mean PR-SRS was 1.4 ± 0.89 (*P* = .102 vs baseline). At 6 months, the mean PR-SRS was 0.8 ± 0.45 (*P* = .038* vs baseline).

After a within-rhPDGF-BB-treated side comparison to baseline, the mean PR-SRS at POD 4 was 0.6 ± 0.55 (*P* = .066 vs baseline). At 1 month, the mean PR-SRS was 1.0 ± 0.00 (*P* = .059 vs baseline). At 3 months, the mean PR-SRS was 1.4 ± 0.89 (*P* = .102 vs baseline). At 6 months, the mean PR-SRS was 0.8 ± 0.45 (*P* = .038* vs baseline).

### Practitioner-Reported PR-SRS

At baseline, the mean practitioner-reported PR-SRS scores were 2.5 ± 0.58 for both the Nanofat- and PDGF-treated hemifaces (*P* = 1.000). At POD 4, the mean scores were 0.00 ± 0.00 for both hemifaces (*P* = 1.000). At 1 month, the mean scores were 0.5 ± 0.58 for the Nanofat-treated hemiface and 0.75 ± 0.50 for the PDGF-treated hemiface (*P* = .317). At 3 months, the mean scores were 0.75 ± 0.50 for the Nanofat-treated hemiface and 1.00 ± 0.00 for the PDGF-treated hemiface (*P* = .317). At 6 months, the mean scores were 0.75 ± 0.50 for both hemifaces (*P* = 1.000).

After a within-Nanofat-treated side comparison to baseline, the mean PR-SRS at POD 4 was 0.00 ± 0.00 (*P* = .038* vs baseline). At 1 month, the mean PR-SRS was 0.5 ± 0.58 (*P* = .039* vs baseline). At 3 months, the mean PR-SRS was 0.75 ± 0.50 (*P* = .041* vs baseline). At 6 months, the mean PR-SRS was 0.75 ± 0.50 (*P* = .059 vs baseline).

After a within-PDGF-treated side comparison to baseline, the mean PR-SRS at POD 4 was 0.00 ± 0.00 (*P* = .038* vs baseline). At 1 month, the mean PR-SRS was 0.75 ± 0.50 (*P* = .034* vs baseline). At 3 months, the mean PR-SRS was 1.00 ± 0.00 (*P* = .038* vs baseline). At 6 months, the mean PR-SRS was 0.75 ± 0.50 (*P* = .059 vs baseline).

### Practitioner-Reported GAIS

**Figure 4.**
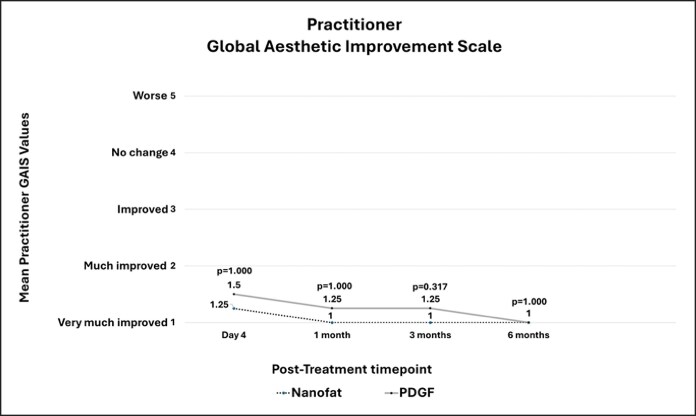
Practitioner Global Aesthetic Improvement Scale (GAIS) Assessment with possible scores of 0 to 5. A score of 1 represents very much improved cosmetic result and a score of 5 represents appearance worse than the original condition. There was no significant difference in GAIS mean scores between the Nanofat- and PDGF-treated sites (all *P* > .05).

At POD 4, the mean GAIS scores were 1.25 ± 0.50 for the Nanofat-treated side and 1.5 ± 1.00 for the PDGF-treated side (*P* = .317; Figure 4). At 1 month, the mean scores were 1.0 ± 0.00 for the Nanofat-treated side and 1.25 ± 0.50 for the PDGF-treated side (*P* = .317). At 3 months, the mean scores were 1.0 ± 0.00 for the Nanofat-treated side and 1.25 ± 0.50 for the PDGF-treated side (*P* = .157). At 6 months, the mean scores were 1.0 ± 0.00 for both sides (*P* = 1.000).

After a within-Nanofat-treated side comparison to POD 4, the mean GAIS at 1 month was 1.0 ± 0.00 (*P* = .317 vs POD 4). At 3 months, the mean GAIS was 1.0 ± 0.00 (*P* = .317 vs POD 4). At 6 months, the mean GAIS was 1.0 ± 0.00 (*P* = .317 vs POD 4). After a within-PDGF-treated side comparison to POD 4, the mean GAIS at 1 month was 1.25 ± 0.50 (*P* = .317 vs POD 4). At 3 months, the mean GAIS was 1.25 ± 0.50 (*P* = 1.000 vs POD 4). At 6 months, the mean GAIS was 1.00 ± 0.00 (*P* = .317 vs POD 4).

### Patient Satisfaction

**Figure 5.**
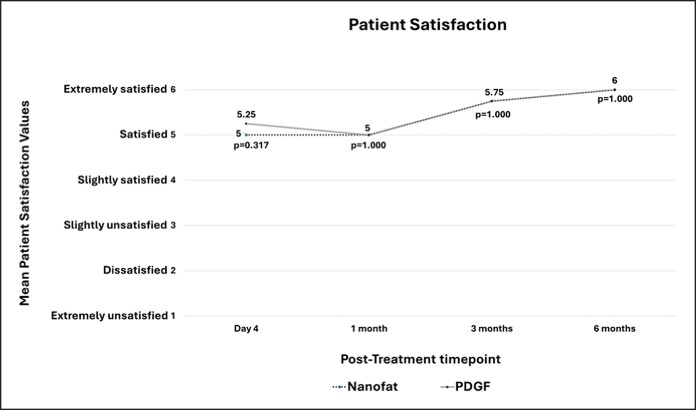
Patient Satisfaction Scores with possible scores of 0 to 6. A score of 1 represents extremely unsatisfied with results and a score of 6 represents extremely satisfied with results. There was no significant difference in patient satisfaction scores between the nanofat- and PDGF-treated sites (all *P* > .05).

**Figure 6.**
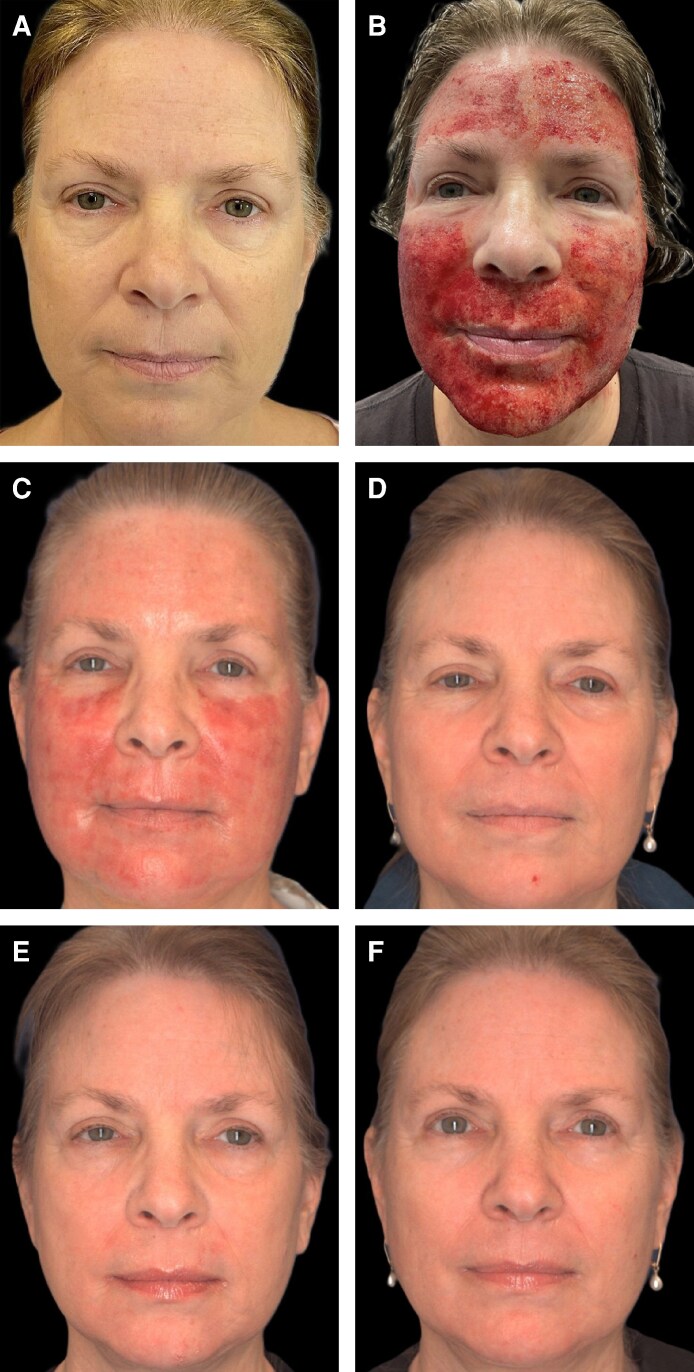
Patient is a 60-year-old female with Fitzpatrick skin type 1. Nanofat-treated side is the left face. Recombinant platelet-derived growth factor–treated side is the right face. (A) Baseline prior to procedure. (B) Immediately after procedure. (C) Postprocedure Day 4. (D) Postprocedure 1 month. (E) Postprocedure 3 months. (F) Postprocedure 6 months.

**Figure 7.**
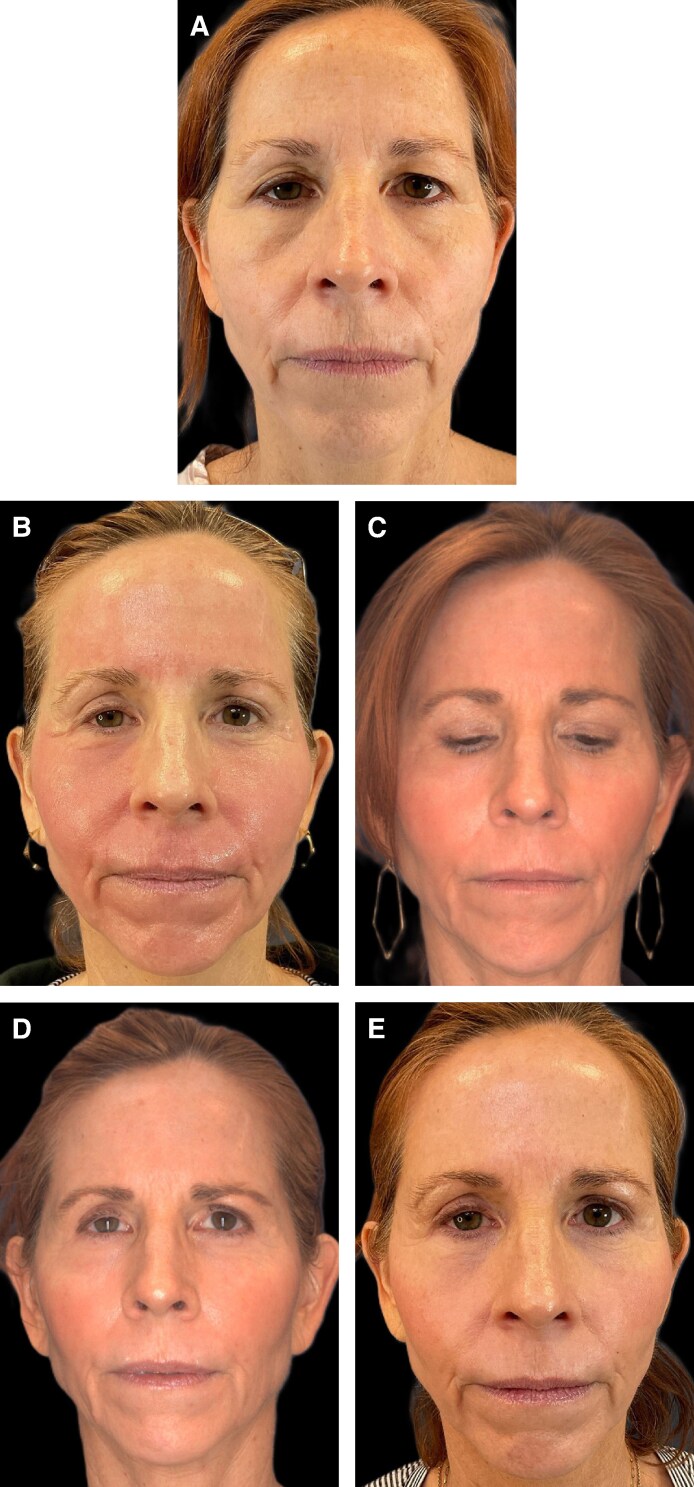
Patient is a 54-year-old female with Fitzpatrick skin type 1. Nanofat-treated side is the right face. Recombinant platelet-derived growth factor–treated side is the left face. (A) Baseline prior to procedure. (B) Postprocedure Day 9. (C) Postprocedure 1 month. (D) Postprocedure 3 months. (E) Postprocedure 6 months.

Patient satisfaction survey had possible scores of 0 (extremely unsatisfied) to 6 (extremely satisfied). At POD 4, the mean satisfaction scores were 5.0 ± 0.82 for the Nanofat-treated hemiface and 5.25 ± 0.50 for the PDGF-treated hemiface (*P* = .317). At 1 month, the mean scores were 5.0 ± 1.41 for both hemifaces (*P* = 1.000). At 3 months, the mean scores were 5.75 ± 0.50 for both hemifaces (*P* = 1.000). At 6 months, the mean scores were 6.0 ± 0.00 for both hemifaces (*P* = 1.000). After a within-nanofat-treated side comparison to POD 4, the mean satisfaction score at 1 month was 5.0 ± 1.41 (*P* = 1.000 vs POD 4). At 3 months, the mean satisfaction was 5.75 ± 0.50 (*P* = .414 vs POD 4). At 6 months, the mean satisfaction was 6.0 ± 0.00 (*P* = .102 vs POD 4) (Figure 5). Figures 6 and 7 show clinical outcomes.

### Histopathology

Prior to treatment, baseline histopathology exhibited an overall thin epidermal layer with flattened rete ridges. The stratum corneum of the epidermis was thickened with hyperkeratosis but with a thinner stratum spinosum. Solar elastosis with minimally dense collagen was present in the dermis.

In the early posttreatment phase, biopsies were collected on Day 4 and 1 patient had the initial postprocedure biopsy on Day 9. On the side treated with the rhPDGF-BB solution, the epidermis showed epidermal necrosis with a pronounced stratum basale and inflammatory infiltrates throughout the dermis with more frequent extravasated erythrocytes and perivascular inflammation. In comparison, the Nanofat-treated side demonstrated similar patterns of injury but exhibited less epidermal eschar, increased thickening of the epidermis, and milder perineural and perivascular infiltrates.

More direct comparisons of the early posttreatment phase showed the Nanofat-treated side of Patient 1 demonstrated focal subepidermal necrosis and minimal perineural inflammation, and the rhPDGF-BB-treated side showed a stronger reaction with acute and chronic inflammation, perivascular inflammation, and areas of subepidermal necrosis. The nanofat-treated side of Patient 2 showed an acute epidermal inflammation and mild-to-moderate dermal inflammation, and the rhPDGF-BB-treated side showed a more pronounced epidermal injury because of focal necrosis. The both sides of Patient 3 showed early postprocedure inflammatory activity, and the rhPDGF-BB showed a more prominent acute inflammatory response compared with the nanofat-treated side. The nanofat-treated side of Patient 4 demonstrated surface hemorrhage with moderate acute and chronic inflammation, and rhPDGF-BB showed loss of the epidermis because of necrosis, abundant extravasated erythrocytes, and perivascular inflammation. Nanofat- and rhPDGF-BB-treated sides of Patient 5 (taken at POD 9) demonstrated mild dermal fibrosis and low-grade inflammatory inflammation, with mild perivascular inflammation.

At 1 month, both the rhPDGF-BB and Nanofat sides showed a compact stratum corneum, thickened epidermal layer, mild dermal neo-collagenesis, and decreased inflammatory infiltrates with a clear progression from acute injury to early remodeling. Trichrome staining showed evenly distributed collagen synthesis in both groups. There is a decrease in solar elastosis with abundant new extracellular matrix and collagen synthesis on both treatment sides. Minimal perineural and peri-adnexal inflammatory infiltrates were present on both treatment sides, although low-grade perivascular inflammation was more commonly retained on the rhPDGF-BB-treated side.

At 3 months posttreatment, both sides had an increase in epidermal thickness, an increase in collagen synthesis with restoration of organized collagen bundles, and a decrease in solar elastosis. Collagen architecture was uniform across groups, adnexal structures remain intact, and no adverse remodeling patterns were observed. The rhPDGF-BB-treated sides had isolated foci of chronic perivascular and perifollicular inflammatory infiltrates compared with the Nanofat-treated side. One sample (Patient 5) was not collected at the 3-month follow-up because the patient declined the biopsy.

Overall, at 6 months, both treatments converged on stable late-stage remodeling, marked by dermal neo-collagenesis, normal adnexal structures, and minimal isolated inflammatory infiltrates. The rhPDGF-BB-treated sides occasionally retained low-grade perivascular and rare perineural inflammation without any evidence of adverse fibrosis. Specifically, the Nanofat-treated side of Patient 1 showed minimal chronic inflammation with mild subepidermal collagen deposition, and the rhPDGF-BB showed mild perivascular inflammation and mild subepidermal collagen deposition, and the overall pattern of regeneration and mild long-term remodeling. The nanofat-treated side of Patient 2 showed minimal perivascular inflammation, and the rhPDGF-BB showed focally moderate acute and chronic inflammatory infiltrates with mild perivascular and perineural inflammation. In Patient 3, both sides demonstrated mild residual inflammation with stable, mature remodeling. The rhPDGF-BB-treated side of Patient 4 demonstrated stable collagen architecture and mild perivascular inflammation (the Nanofat side could not be fully assessed because of artifact). The Nanofat-treated side of Patient 5 showed new collagen synthesis consistent with late-stage remodeling, and the rhPDGF-BB-treated side showed occasional mild isolated foci of chronic perivascular inflammation and mild perineural inflammation.

**Figure 8.**
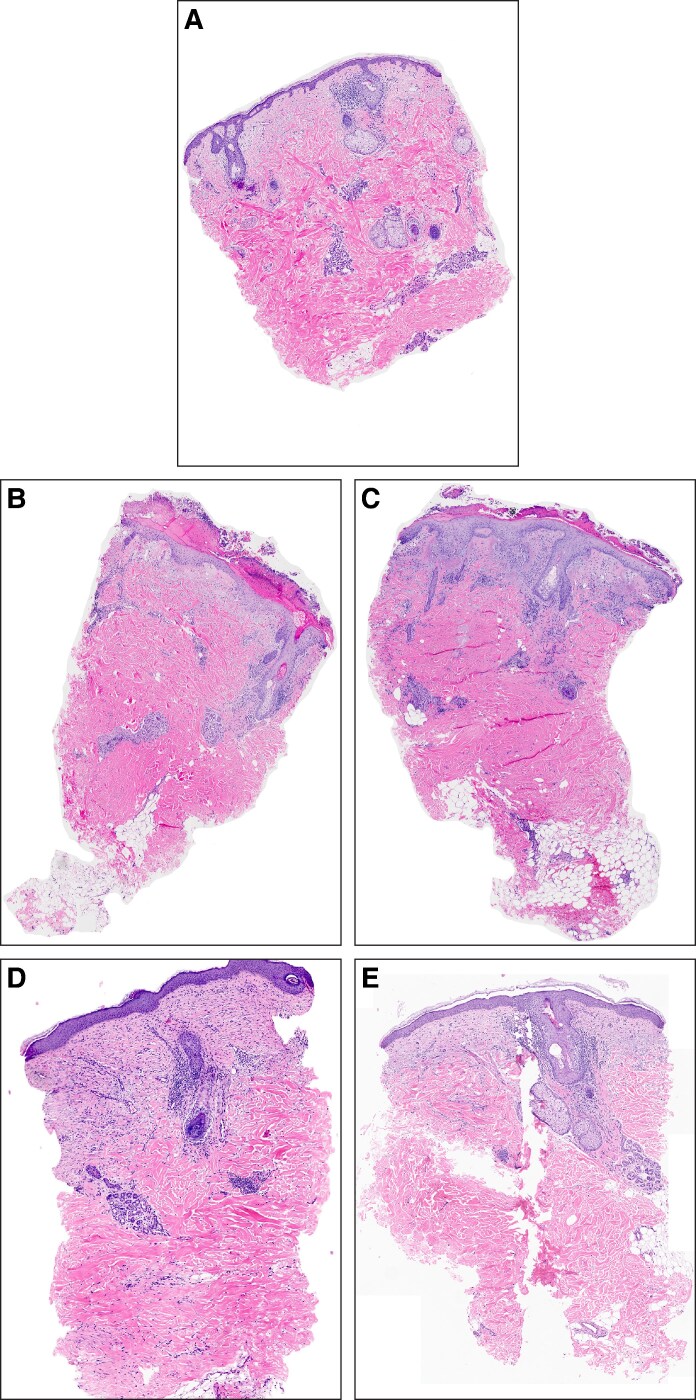

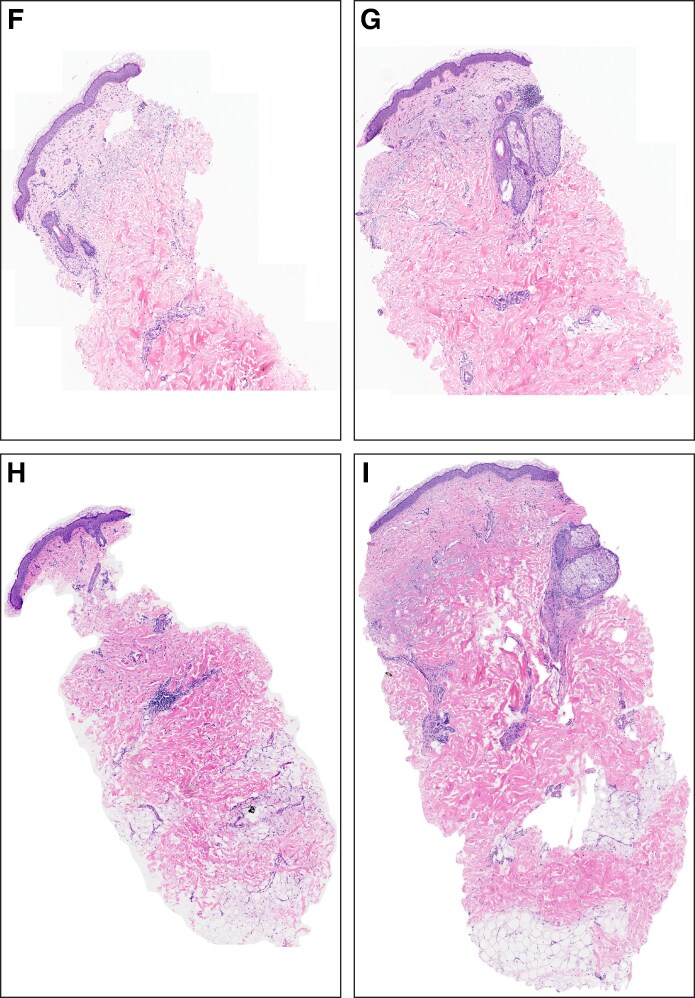
Histopathological analysis of autologous nanofat and recombinant platelet-derived growth factor (rhPDGF-BB) in a 68-year-old female. (A) Baseline prior to treatment. (B) Autologous nanofat at postprocedure Day 4. (C) rhPDGF-BB at postprocedure Day 4. (D) Autologous nanofat after 1 month. (E) rhPDGF-BB after 1 month. (F) Autologous nanofat after 3 months. (G) rhPDGF-BB after 3 months. (H) Autologous nanofat after 6 months. (I) rhPDGF-BB after 6 months.

All histology slides from Patient 3 are shown in Figure 8. All qualitative descriptions by a third-party dermatopathologist of histological H&E and trichrome slides are included in Supplemental Table 1.

## DISCUSSION

Autologous Nanofat is the gold standard “rescue” for mechanical and thermal injury for skin rejuvenation.^22,23,29-33^ Derived from lipoaspirate, autologous Nanofat is enriched with a stromal vascular fraction, which contains ASCs, mesenchymal progenitors, leukocytes, pericytes, and vascular smooth muscle cells.^11,12,14^ These cell populations have been shown to secrete cytokines and growth factors that can stimulate angiogenesis, fibroblast proliferation, extracellular matrix remodeling, and immunomodulation.^13,34^ These processes are vital to wound healing, especially after thermal and mechanical trauma to tissue. This was supported, clinically and histologically, by Claytor et al in a retrospective study evaluating the healing capability of autologous Nanofat “rescue” following fractional CO_2_ laser and mechanical microneedling tissue trauma, which demonstrated that Nanofat significantly reduced posttreatment pain and accelerated wound healing with superior and long-lasting final results. Prior to this, the concurrent use of CO_2_ laser and mechanical microneedling was uncommon, largely owing to the potential for additive adverse side effects.^27^

To our knowledge, this is the first split-face, patient-blinded, randomized controlled trial to study the safety and efficacy of rhPDGF-BB compared to autologous Nanofat following CO_2_ laser and mechanical microneedling on facial skin. The results of this trial demonstrate that rhPDGF-BB serves as a safe and effective rescue to combined CO_2_ laser and mechanical microneedling for perioral and facial skin rejuvenation. Histopathologic evaluation confirmed re-epithelialization and abundant extracellular matrix formation with both autologous Nanofat and rhPDGF-BB treatments by 6 months. Patient- and practitioner-reported outcomes showed sustained improvement in rhytid severity (PR-SRS), global aesthetic appearance (GAIS), and high satisfaction through follow-up. This study suggests that rhPDGF-BB is a safe, effective, and less invasive alternative, and potentially a time-saving enhancement, in regenerative aesthetic medicine to target facial rhytids.

Although PRP may have regenerative capacity, results are often variable. Recombinant pure PDGF offers the advantages of delivering a predictable concentration of growth factors at much higher levels. Natural PDGF-BB is a 24 to 30 kDa protein secreted by degranulation of alpha granules in activated platelets during tissue injury and is the most potent regenerative isoform found in the body.^19,35^ rhPDGF-BB is a pharmaceutically produced replica of natural PDGF-BB and has been proven to have potent chemotactic, mitogenic, and angiogenic properties. To date, such properties have been primarily harnessed to promote the healing of chronic skin wounds and regenerate tissues following oral and maxillofacial and orthopedic surgery.^18,19,36^ As a result, rhPDGF-BB has received 4 FDA approvals for wound healing and tissue regeneration indications.^37-40^ Our results further confirm the efficacy of rhPDGF-BB in wound healing, and in particular, its clinical utility in the field of aesthetic medicine.

Although use of rhPDGF-BB in skin resurfacing is novel in medical aesthetics, this biologic has an established record of safe and effective use in wound healing.^18,19,38-43^ Steed evaluated the effect of the topical application of rhPDGF-BB on chronic lower extremity skin wounds in patients with diabetes mellitus and found that wounds healed completely on average 6 weeks earlier compared with placebo.^44^ Pierce et al showed that rhPDGF-BB accelerated healing in patients with chronic pressure ulcers, as well as its capability to upregulate innate immune cell chemotaxis and improve matrix synthesis after topical application on excisional wounds.^43^  ^,45^ Shackelford et al found that using topical rhPDGF-BB on abdominal wound separation resulted in closure 24 days earlier compared with placebo, thus suggesting that rhPDGF-BB can accelerate the wound-healing process.^46^ Hom and Manivel demonstrated improved wound healing in chronic irradiated neck lesions after the application of rhPDGF-BB for 6 months, with tissue biopsy revealing thickening of the epidermis and papillary dermis, increased vascularity, and inflammatory infiltrates.^47^ Similarly, Uhl et al showed that rhPDGF-BB-treated ischemic tissue had significantly improved vascularization and immune cell presence.^48^ Data from the current study are consistent with the previous literature. Specifically, the use of rhPDGF-BB in our study lessened the inflammatory cascade associated with CO_2_ laser treatment while also stimulating collagen production and skin rejuvenation.

Ablative CO_2_ laser and mechanical microneedling induce an inflammatory response in tissue because of localized injury in the epidermis and dermis, activating the chemotaxis of lymphocytes, macrophages, and neutrophils to initiate the wound-healing process.^6,7,49^ To study the comparative results between rhPDGF-BB treatment and autologous Nanofat, rhPDGF-BB or autologous Nanofat was topically applied immediately after laser resurfacing and microneedling. We found similar clinical results between the 2 groups. Tissue biopsies were obtained from the posterior auricular region treated with CO_2_ ablative laser and microneedling followed by rescue autologous Nanofat or rhPDGF-BB. On histopathology, autologous Nanofat and rhPDGF-BB each demonstrated improvement in healing trajectories and extracellular matrix production, with only subtle differences mostly related to transient and mild inflammatory changes. Autologous Nanofat showed consistently milder inflammatory response by POD 4 and rhPDGF-BB showing mild isolated foci of inflammatory infiltrates. Patient surveys demonstrated comparable satisfaction and reduction of perioral rhytids by 6 months for both groups. Thus, rhPDGF-BB may result in amplified chemotaxis, angiogenesis, and extracellular matrix remodeling in skin similar to autologous Nanofat following thermal (CO_2_ laser) and mechanical microneedling injury. By 6 months, this study showed that both treatment groups demonstrated on histology re-epithelialization with a thin and compact stratum corneum, thickened stratum spinosum, and increased collagen synthesis, resembling more youthful skin architecture.

By 1 month after treatment, both the rhPDGF-BB and autologous Nanofat-treated sides showed comparable collagen and extracellular matrix formation and complete re-epithelialization. The tissues continued to undergo normal physiologic remodeling at 3 and 6 months. No adverse reactions were present histologically on either side; likewise, there was no evidence of tumorigenic or hyperproliferation observed, and no overexpression of collagen or other extracellular matrices. Overall, both autologous nanofat- and rhPDGF-BB-treated sides supported normal wound healing and produced comparable tissue remodeling over time.

It should be emphasized that only pure rhPDGF-BB aqueous solution was applied on the rhPDGF-BB-treated side in this study. The sterile HA serum that is packaged with the rhPDGF-BB in the commercial product (Ariessence Pure PDGF+) was not used to allow a strict comparison of the rhPDGF-BB aqueous solution to Nanofat and avoid confounding interpretation that could have been introduced by mixing the rhPDGF-BB with the HA as recommended by the manufacturer.

Despite the strengths of this study, there are limitations worth considering. This study included a small sample size of 5 females, which limited statistical power and generalizability. The practitioner was not blinded to treatment allocation, which may introduce bias; however, the practitioner performed the procedure and administered the study treatments. In order to balance this, the histopathology analysis was conducted by a third-party, blinded company. This study lacked a true control group, as autologous Nanofat was compared to rhPDGF-BB rather than a “no-treatment” group. Future studies with larger sample sizes are needed to continue studying the clinical and histological impact of autologous Nanofat and/or rhPDGF-BB for skin rejuvenation in comparison to a no-treatment group. The pain scores collected for the face were a global score rather than pain scores assigned to each hemiface which limits the comparison between both treatment groups. Moreover, studies are needed that evaluate the impact of rhPDGF-BB on various Fitzpatrick skin types. A future study evaluating the combination of Nanofat and rhPDGF-BB would be of significant interest.

## CONCLUSIONS

This pilot, split-face, patient-blinded randomized controlled study demonstrates that rhPDGF-BB serves as a safe and effective rescue to combined CO_2_ laser and mechanical microneedling for perioral and facial skin rejuvenation when compared to autologous Nanofat. Histopathologic evaluation confirmed re-epithelialization and abundant extracellular matrix formation with both autologous Nanofat and rhPDGF-BB treatments by 6 months. Patient- and practitioner-reported outcomes showed sustained improvement in rhytid severity, global aesthetic appearance, and high satisfaction through follow-up. This study suggests that rhPDGF-BB can serve as a safe and effective alternative in regenerative aesthetic medicine to target facial rhytids and, taken together, provides early insight into the clinical potential of rhPDGF-BB in wound healing and facial skin rejuvenation in the field of aesthetic medicine.

## Supplemental Material

This article contains supplemental material located online at https://doi.org/10.1093/asjof/ojag033.

## Supplementary Material

ojag033_Supplementary_Data
