# Supplementary material for: Recombinant Platelet-Derived Growth Factor BB vs Autologous Nanofat to Enhance Recovery After CO2 Laser and Microneedling: A Split-Face, Randomized Controlled Trial
Source: Aesthet Surg J Open Forum. 2026 Mar 6;8:ojag033. doi: 10.1093/asjof/ojag033 (PMC13015916; doi:10.1093/asjof/ojag033)

**Appendix A.** Patient-reported surveys consisted of satisfaction scores and the Perioral Rhytids Severity Rating Scale (PR-SRS). Baseline assessment included PR-SRS only, with subsequent assessments of both surveys conducted at post-procedure day 4 and at 1-, 3-, and 6-month follow-up visits.


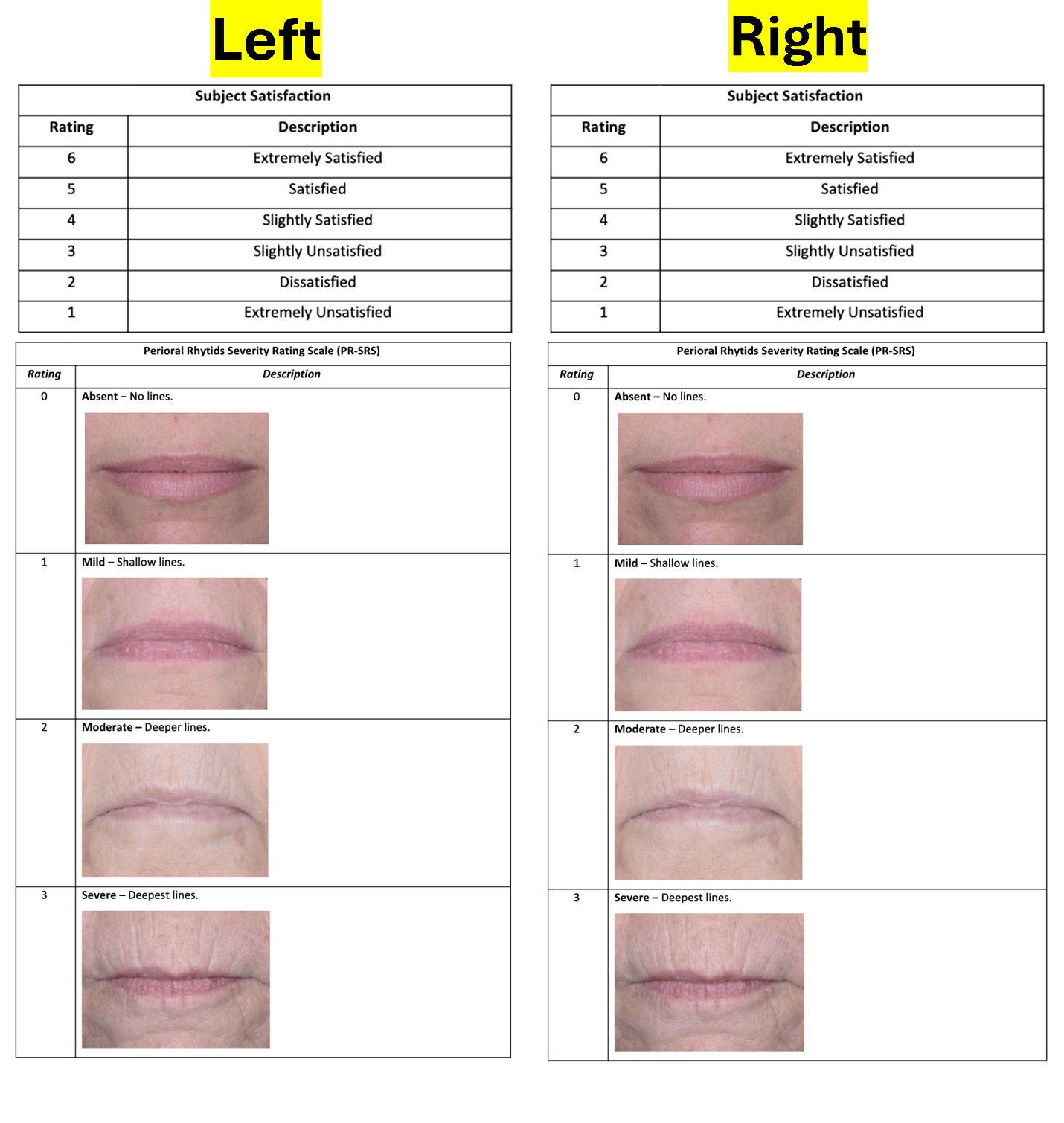

Supplement: ojag033_Supplementary_Data [file ojag033_supplementary_data.zip › Appendix A.docx]
