# Supplementary material for: Recombinant Platelet-Derived Growth Factor BB vs Autologous Nanofat to Enhance Recovery After CO2 Laser and Microneedling: A Split-Face, Randomized Controlled Trial
Source: Aesthet Surg J Open Forum. 2026 Mar 6;8:ojag033. doi: 10.1093/asjof/ojag033 (PMC13015916; doi:10.1093/asjof/ojag033)
Supplement: ojag033_Supplementary_Data [file ojag033_supplementary_data.zip › Appendix B.docx]

**Appendix B.** Practitioner-reported outcome surveys included the Global Aesthetic Improvement Scale (GAIS) and the Perioral Rhytids Severity Rating Scale (PR-SRS). Baseline assessment included PR-SRS only, with both surveys completed at post-procedure day 4 and at 1-, 3-, and 6-month follow-up visits.

| Left | Right |
| --- | --- |
| 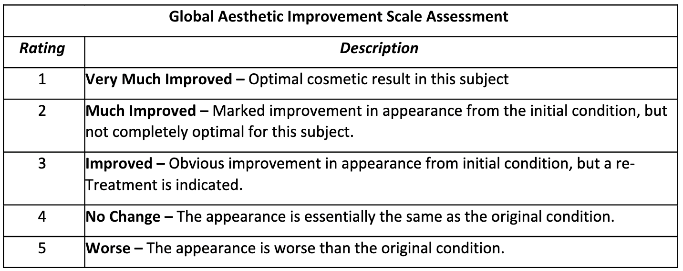 | 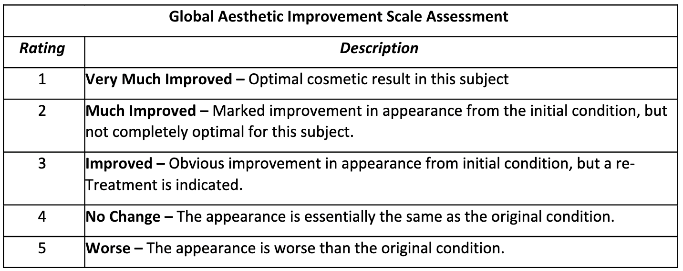 |
| 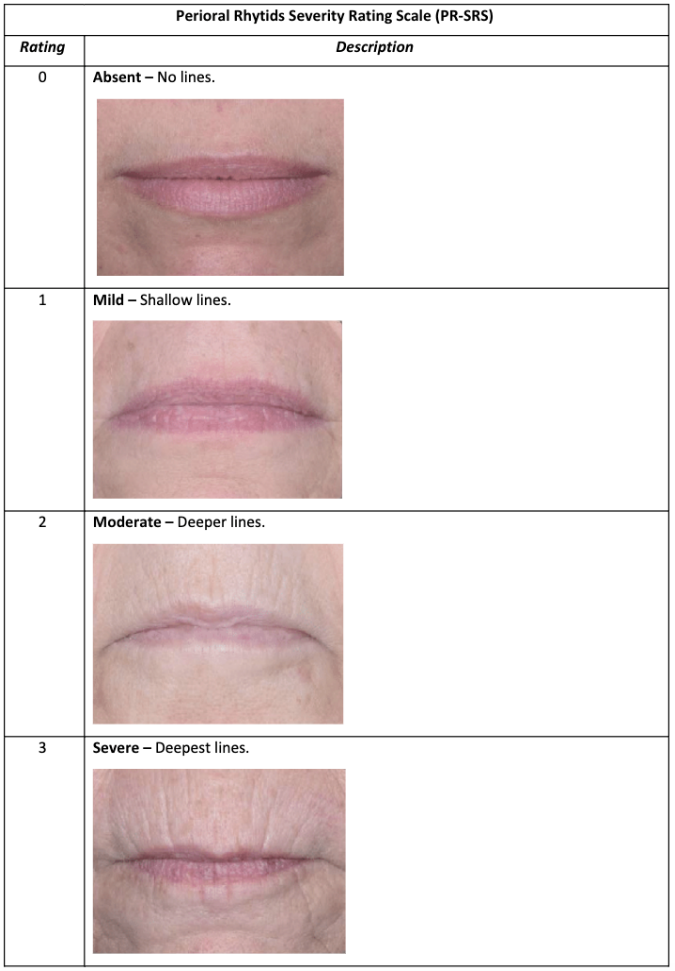 | 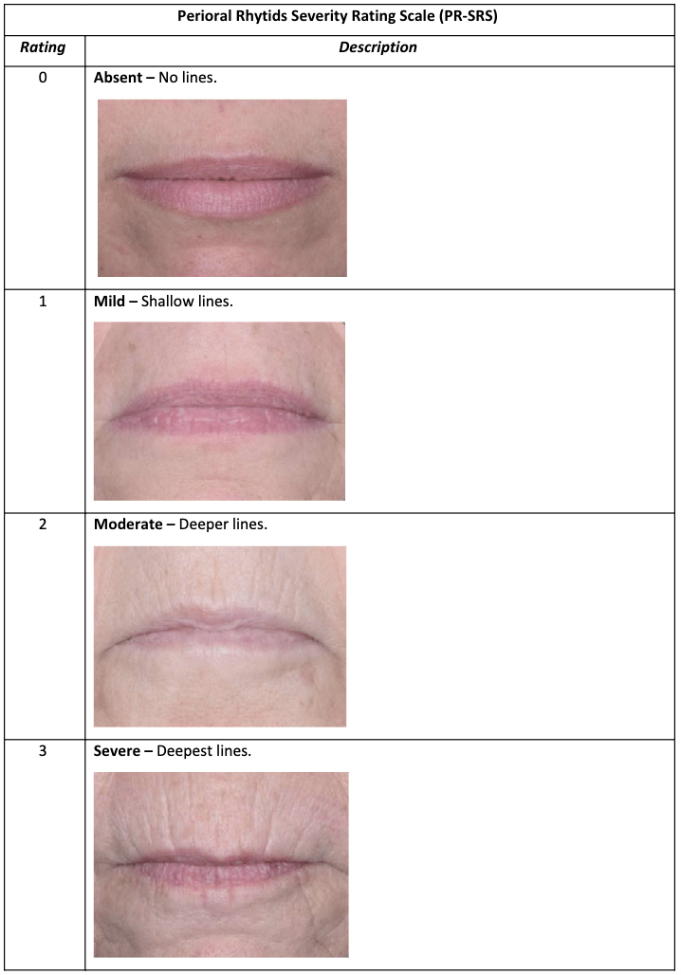 |
