# Supplementary material for: Recombinant Platelet-Derived Growth Factor BB vs Autologous Nanofat to Enhance Recovery After CO2 Laser and Microneedling: A Split-Face, Randomized Controlled Trial
Source: Aesthet Surg J Open Forum. 2026 Mar 6;8:ojag033. doi: 10.1093/asjof/ojag033 (PMC13015916; doi:10.1093/asjof/ojag033)
Supplement: ojag033_Supplementary_Data [file ojag033_supplementary_data.zip › Supplemental Table 1.docx]

**Supplemental Table 1**. Qualitative Histopathologic Assessment of Facial Skin Biopsy Samples Following Split-face Treatment With Autologous Nanofat or rhPDGF-BB

| Patient | Baseline v. POD 4 | POD 4 v. 1 month | 1 month v. 3 months | 3 months v. 6 months | Overall |
| --- | --- | --- | --- | --- | --- |
| Patient 1 | Compared to baseline, both treatment sides show expected acute post-procedure changes at POD 4. The nanofat side demonstrates focal subepidermal necrosis, minimal perineural inflammation, and minimal early fibrosis, while adnexal structures remain intact. The PDGF-treated side shows a stronger reaction in regions readable on H&E, including moderate to marked acute and chronic inflammation, perivascular inflammation, moderate dermal fibrosis, and areas of subepidermal necrosis. Two PDGF H&E slides are limited by folding, but the evaluable areas consistently show a more prominent inflammatory and early fibrotic response than nanofat. | Between POD 4 and 1 month, both treatment sides transition from acute injury to early remodeling. Nanofat-treated skin shows mild subepidermal fibrosis, mild chronic inflammation, and no perineural inflammation or red blood cell extravasation, with trichrome demonstrating organized mild collagen and early maturation. The PDGF-treated side shows a reduction from moderate acute injury to minimal–mild fibrosis with mild chronic inflammation, and trichrome reveals evenly distributed, lightly compact collagen. Overall, inflammation decreases, and collagen organization improves in both groups by this timepoint. | From 1 to 3 months, both treatment sides continue toward stable, mild remodeling patterns. Nanofat-treated tissue shows diffusely mild fibrosis and minimal chronic inflammation, with trichrome demonstrating evenly distributed mild collagen. PDGF-treated tissue shows a similar pattern with diffusely mild fibrosis, mild perivascular inflammation, and features described as suggestive of regeneration. Nanofat and PDGF look very similar at this stage, with no major qualitative differences in fibrosis or inflammatory features. | By 6 months, both treatment groups show stable late-stage remodeling characterized by mild subepidermal fibrosis and low-grade inflammation. Nanofat-treated tissue shows minimal chronic inflammation and diffusely mild fibrosis, with trichrome confirming mild subepidermal collagen deposition. The PDGF-treated side shows mild subepidermal fibrosis with mild chronic and mild perivascular inflammation, and the overall pattern remains suggestive of regeneration. Both treatments show mild, stable long-term remodeling. | Across all timepoints, Patient 1 demonstrates a consistent remodeling pattern under both treatments. After an early period of acute injury at POD 4, particularly on the PDGF side, both treatment arms progress to mild, organized fibrosis by 1 month. At 3 and 6 months, nanofat and PDGF converge into similar mild remodeling patterns with preserved adnexal structures and minimal residual inflammation, apart from relatively higher perivascular inflammation in the PDGF-treated side. Both treatments ultimately show mild, stable long-term histologic changes. |
| Patient 2 | Both treatment sides show expected acute post-procedure changes at POD 4. The nanofat-treated side demonstrates a thin but cellular epidermis with focal hyperkeratosis, acute epidermal inflammation, mild to moderate dermal inflammation, mild dermal fibrosis, and perivascular inflammation. A detached necrotic fragment is present. The PDGF-treated side shows a very thin epidermis with focal acute inflammation, mild acute and chronic dermal inflammation, mild dermal fibrosis, and focal epidermal necrosis. Mild perivascular inflammation is noted. Overall, both sides demonstrate an active early inflammatory reaction with mild fibrosis. The PDGF-treated side shows more pronounced epidermal injury due to focal necrosis, while nanofat shows a somewhat broader inflammatory profile involving the epidermis and superficial dermis | At 1 month, both treatment arms show a transition from acute inflammation to early remodeling. The nanofat-treated side shows mild perifollicular acute and chronic inflammation, mild dermal fibrosis, and focal hyperkeratosis. The collagen pattern is mildly fibrotic and compatible with early healing. The PDGF-treated side shows moderate perifollicular inflammation, mild dermal fibrosis, and mild perivascular inflammation. Trichrome confirms mild fibrosis with evenly distributed collagen. Both sides demonstrate early remodeling with mild fibrosis. PDGF-treated tissue retains slightly more inflammatory activity at this timepoint, while nanofat shows a more subdued inflammatory profile with mild fibrosis | By 3 months, both treatment sides continue toward stable intermediate-stage remodeling. The nanofat side shows mild acute and chronic inflammation, mild dermal fibrosis, and mild perivascular inflammation. Trichrome demonstrates mild to moderate dermal fibrosis, with subepidermal collagen denser than in other nanofat cases. The PDGF side shows focally moderate acute and chronic inflammation, mild dermal fibrosis, moderate perivascular inflammation, and mild perineural inflammation. These findings represent a somewhat more active inflammatory pattern compared with the nanofat side. Both sides show stable fibrosis and reduced acute inflammation by this interval, but PDGF retains a higher degree of inflammation than nanofat | By 6 months, both sides show stable late-stage remodeling. The nanofat-treated side shows mild dermal fibrosis and minimal perivascular inflammation. The PDGF-treated side shows mild dermal fibrosis with focally moderate acute and chronic inflammation around a hair follicle, along with mild perivascular and perineural inflammation. Trichrome indicates mild fibrosis with less dense collagen. Both treatments show mild, stable fibrosis at this later interval. The nanofat side demonstrates a quieter inflammatory pattern, while PDGF continues to show low-grade but persistent perivascular and focal inflammatory activity. | Across all timepoints, Patient 2 demonstrates treatment-related remodeling patterns on both the nanofat and PDGF sides. Both sides show expected acute inflammatory changes at POD 4, transitioning to mild fibrosis and reduced inflammation at 1 month. At 3 months, nanofat shows mild and stable remodeling, while PDGF demonstrates a somewhat more active inflammatory pattern with moderate perivascular involvement. By 6 months, both arms show stable late-stage remodeling with mild fibrosis. Nanofat displays minimal residual inflammation, whereas PDGF retains low-grade perivascular and focal follicular inflammation. Overall, both treatment modalities show consistent remodeling and stable fibrosis. Nanofat-treated tissue generally demonstrates a quieter inflammatory profile, while PDGF-treated tissue shows mild but more persistent inflammatory features across later intervals |
| Patient 3 | Compared to baseline, both treatment sides show clear early post-procedure inflammatory changes at POD4. The baseline samples demonstrate a thin but cellular epidermis with a single inflamed hair shaft and otherwise unremarkable adnexal structures, along with mild chronic inflammation. Trichrome shows decreased collagen density beneath the epidermis with mild dermal fibrosis, establishing the collagen reference pattern for this patient. At POD4, the Nanofat-treated skin shows hyperkeratosis with intraepidermal, subcorneal, and surface acute inflammation, including areas of intraepidermal necrosis. The dermis contains foci of acute and chronic inflammation associated with a hair shaft and sebaceous gland, along with mild perivascular inflammation and mild fibrosis. The PDGF-treated side also shows hyperkeratosis, with necrosis of the stratum corneum and diffuse moderate acute and chronic inflammation around hair shafts and sebaceous glands. Moderate perivascular inflammation and extravasation of red blood cells are present, with mild dermal fibrosis. Both sides reflect expected early post-treatment inflammatory activity, with the PDGF region showing a more prominent acute inflammatory response than the Nanofat region. | Between POD4 and 1 month, both treatment sides transition from acute injury patterns to early remodeling. The Nanofat side shows a cellular epidermis with mild dermal fibrosis and mild focal acute and chronic inflammation near hair shafts, along with mild to moderate perivascular inflammation. The PDGF-treated sample demonstrates a cellular epidermis with mild dermal fibrosis and moderate acute and chronic inflammation surrounding a hair follicle. Sebaceous and eccrine glands remain unremarkable. Both slides reflect early remodeling changes at one month, with PDGF maintaining a slightly stronger inflammatory component compared with Nanofat | From 1 to 3 months, both treatment sides continue into intermediate-stage remodeling. Nanofat-treated tissue shows a cellular epidermis with mild dermal fibrosis and unremarkable adnexal structures. Trichrome confirms mild fibrosis with less dense collagen beneath the epidermis. The PDGF-treated side shows mild dermal fibrosis with mild acute and chronic inflammation near clusters of follicles and sebaceous glands. Trichrome demonstrates mild fibrosis with collagen density approaching that of the deeper dermis, reflecting a slightly more pronounced fibrotic response than the Nanofat side. Overall, both treatment groups display similar qualitative intermediate-stage remodeling patterns | By 6 months, both treatment groups show stable late-stage remodeling. The Nanofat sample contains a cellular epidermis with mild dermal fibrosis and focal mild acute and chronic inflammation in the deeper dermis. Sampling is narrow, but findings are consistent with late-stage maturation. The PDGF-treated tissue shows a cellular epidermis with mild dermal fibrosis, unremarkable hair shafts, sebaceous glands, and eccrine glands, and minimal chronic inflammation near an apocrine gland. Trichrome shows mild fibrosis with less dense collagen, consistent with late-stage remodeling. At this interval, both sides demonstrate stable, mature remodeling with only mild residual inflammation | Across all timepoints, Patient 3 demonstrates consistent remodeling on both nanofat- and PDGF-treated sides. At POD 4, both sides show acute post-procedure inflammatory changes, with nanofat showing prominent epidermal injury and focal necrosis, while PDGF demonstrates a stronger dermal inflammatory response with moderate perivascular inflammation and extravasated red blood cells. By 1 month, both sides transition to mild dermal fibrosis with decreasing inflammation, though the nanofat side shows slightly more perivascular activity, and the PDGF side shows moderate perifollicular inflammation. At 3 months, nanofat demonstrates stable mild fibrosis with preserved adnexal structures, while PDGF shows slightly denser subepidermal collagen on trichrome but maintains a similar overall pattern of intermediate-stage remodeling. By 6 months, both treatments show mild, stable late-stage fibrosis with minimal residual inflammation and normal adnexal structures. Overall, both treatment arms follow parallel healing trajectories, with PDGF showing modestly greater inflammatory intensity at earlier intervals but converging with nanofat by late remodeling. |
| Patient 4 | Compared with baseline, both treatment areas at POD 4 show clear acute post-procedure injury. The Nanofat-treated side demonstrates surface hemorrhage with moderate acute and chronic inflammation and mild dermal fibrosis. The PDGF-treated side shows a more pronounced injury pattern, including loss of the epidermis due to necrosis, abundant extravasated red blood cells, and moderate perivascular inflammation. Collagen on trichrome is mildly less dense on the Nanofat side but appears denser and more fibrotic on the PDGF side at this early interval | By 1 month, both treatment slides show resolution of the marked acute inflammatory findings seen at POD 4. The Nanofat and PDGF samples each demonstrate a cellular epidermis with mild dermal fibrosis and unremarkable adnexal structures. Inflammation is greatly reduced compared with POD 4. Trichrome findings show mild fibrosis with less-dense collagen on both treatment sides, consistent with early remodeling. No significant differences are observed between Nanofat and PDGF at this stage | At 3 months, the Nanofat-treated samples show minimal to mild subepidermal fibrosis, consistent with progression toward intermediate remodeling. The PDGF-treated samples also demonstrate mild dermal fibrosis but show even less fibrosis than the Nanofat samples at the same interval, along with mild chronic perivascular inflammation. Collagen remains mildly or minimally dense on trichrome for both treatment types. Overall, both sides show stable intermediate-stage remodeling, with the PDGF side demonstrating a slightly less fibrotic profile at this timepoint | At 6 months, evaluation of the Nanofat side is limited because the epidermis is cut through on both H&E and trichrome, preventing assessment of dermal fibrosis. Adnexal structures present are unremarkable. On the PDGF side, the epidermis is intact, and there is minimal, if any, dermal fibrosis with mild perivascular inflammation. Trichrome shows subepidermal collagen density similar to that of the deeper dermis, consistent with stable late-stage remodeling. Findings suggest a quiet, minimally fibrotic pattern at this interval on the PDGF-treated side. | Across all timepoints, Patient 4 demonstrates expected treatment-related changes on both the Nanofat- and PDGF-treated sides. POD 4 shows the most pronounced acute injury, with the PDGF side demonstrating greater epidermal loss, hemorrhage, and early inflammatory change compared with Nanofat. By 1 month, both treatment areas transition to mild dermal fibrosis with reduced inflammation and unremarkable adnexal structures. At 3 months, both sides show mild intermediate-stage remodeling, with the PDGF side displaying slightly less fibrosis than Nanofat at the same interval. By 6 months, the PDGF side demonstrates minimal fibrosis with stable collagen architecture and mild perivascular inflammation, while the Nanofat side cannot be fully assessed due to artifact. Overall, both treatments show consistent remodeling patterns, with the PDGF-treated site trending toward a slightly less fibrotic profile at later intervals |
| Patient 5 | At baseline, the dermis shows mild fibrosis with less-dense collagen beneath the epidermis, and adnexal structures appear unremarkable. By POD 9, both Nanofat- and PDGF-treated samples demonstrate expected early post-treatment inflammatory changes, including mild dermal fibrosis and low-grade chronic inflammation. Mild perivascular inflammation is present on both sides. Overall, both treatment arms show similar early injury and remodeling patterns relative to baseline. | By 1 month, Nanofat-treated tissue shows mild dermal fibrosis and a cellular epidermis, with unremarkable adnexal structures. The inflammatory component decreases compared with POD 9, though mild perivascular inflammation remains. PDGF-treated samples at 1 month show mild subepidermal fibrosis and mild perivascular inflammation, though one section has limited dermis, reducing evaluability. Trichrome confirms mild fibrosis on both sides. Overall, both treatment arms move from early inflammatory changes at POD 9 toward mild, more organized remodeling at 1 month. | N/A  No 3-month samples | At 6 months, Nanofat-treated tissue shows stable mild fibrosis with unremarkable adnexal structures, consistent with late-stage remodeling. PDGF-treated samples also demonstrate mild dermal fibrosis with persistent mild chronic perivascular inflammation and mild perineural inflammation. Across both treatments, fibrosis remains mild, and inflammatory features are low-grade. The trichrome stain for the PDGF 6-month sample is technically inadequate but partially suggests collagen density similar to the H&E interpretation. Overall, both sides show stable remodeling patterns at this late interval. | Across all timepoints, Patient 5 demonstrates mild remodeling patterns under both Nanofat and PDGF treatments. Baseline tissue shows mild dermal fibrosis with less-dense collagen beneath the epidermis. By POD 9, both treatment arms display expected early post-procedure changes, including mild fibrosis and low-grade inflammation, with mild perivascular inflammation identified on both sides. At 1 month, fibrosis remains mild and more organized, and inflammatory features decrease, though mild perivascular inflammation persists in both treatment arms. By 6 months, both Nanofat- and PDGF-treated tissues show stable mild fibrosis with unremarkable adnexal structures; the PDGF side retains mild chronic perivascular and perineural inflammation. Overall, both treatment modalities demonstrate comparable patterns of mild fibrosis, but the peri-vascular inflammation seems to be relatively higher in PDGF-treated side. |

For each patient (patient 1 – 5), punch biopsy specimens were obtained from the postauricular region at baseline and at post-procedure (POD) day 4, 1 month, 3 months, and 6 months following treatment. Histologic evaluation was performed on hematoxylin and eosin (H&E) and Masson trichrome stained sections. Each column summarizes qualitative features observed at the indicated timepoint. Descriptors represent comparative, qualitative assessments between the autologous Nanofat and rhPDGF-BB treated sides within each patient. Histopathological analysis was conducted by a third-party, blinded board-certified dermatopathologist (Lanterne Dx, Boulder, CO).
